# Supplementary material for: Preparation and Characterization of Photocatalytically Active Antibacterial Surfaces Covered with Acrylic Matrix Embedded Nano-ZnO and Nano-ZnO/Ag
Source: Nanomaterials (Basel). 2021 Dec 14;11(12):3384. doi: 10.3390/nano11123384 (PMC8703771; doi:10.3390/nano11123384)
Supplement: Supplementary file 1 [file nanomaterials-11-03384-s001.zip › nanomaterials-1480289-supplementary.pdf]

nanomaterials

Supplementary Materials

Preparation and Characterization of Photocatalytically Active Antibacterial Surfaces Covered with Acrylic Matrix Embedded Nano-ZnO and Nano-ZnO/Ag

Merilin Rosenberg [1,2,3], Meeri Visnapuu [4], Kristjan Saal [4], Dmytro Danilian [4], Rainer Pärna [4], Angela Ivask [1,*] and Vambola Kisand [4,*]

1 Institute of Molecular and Cell Biology, University of Tartu, Riia 23, 51010 Tartu, Estonia; merilin.rosenberg@ut.ee (M.R.)
2 Laboratory of Environmental Toxicology, National Institute of Chemical Physics and Biophysics, Akadeemia tee 23, 12618 Tallinn, Estonia
3 Department of Chemistry and Biotechnology, Tallinn University of Technology, Akadeemia tee 15, 12618 Tallinn, Estonia
4 Institute of Physics, University of Tartu, W. Ostwaldi Str 1, 50411 Tartu, Estonia; meeri.visnapuu@ut.ee (M.V.); kristjan.saal@ut.ee (K.S.); dmytro.danilian@ut.ee (D.D.); rainer.parna@ut.ee (R.P.)
* Correspondence: angela.ivask@ut.ee (A.I.); vambola.kisand@ut.ee (V.K.)

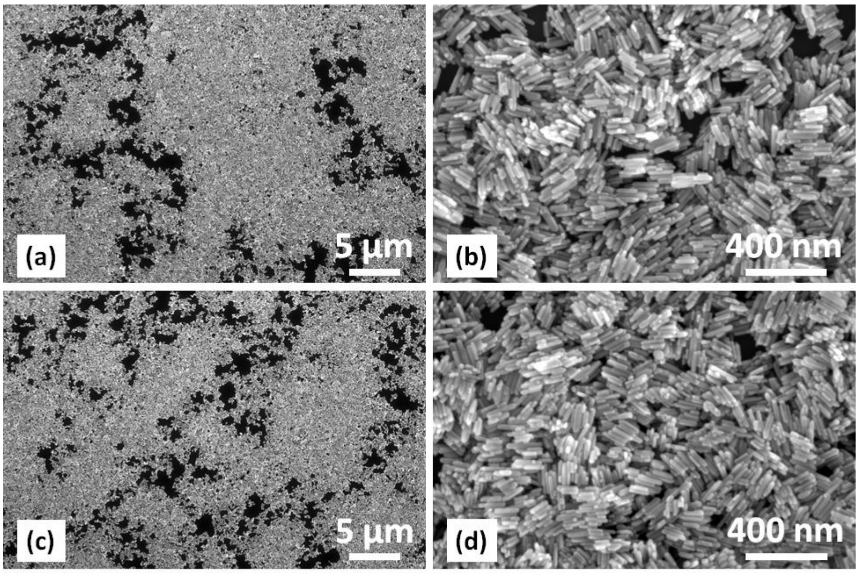

Figure S1. SEM images of nano-ZnO (**a, b**) and nano-ZnO/Ag (**c, d**) covered silicon surfaces. Images show a similar coverage of substrates by bare nanoparticles.

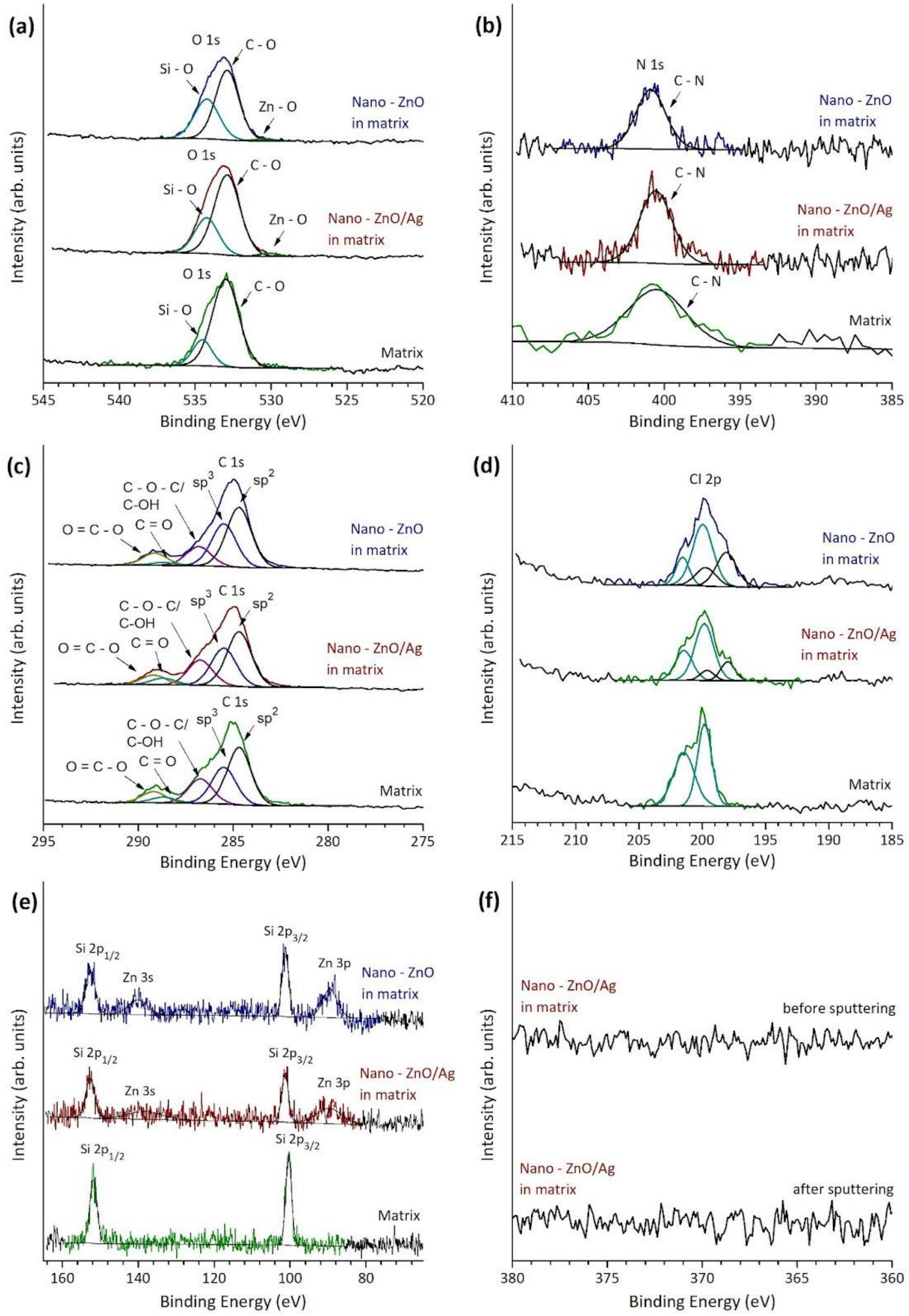

Figure S2. (a) The O 1s, **(b)** N 1s, **(c)** C 1s, **(d)** Cl 2p and **(e)** Si 2p XPS spectra (hν = 1486.6 eV) of nano-ZnO, nano-ZnO/Ag containing coating and pure matrix material on wood substrate. **(f)** Spectral region respective to Ag 3d photoline of nano-ZnO/Ag containing coating on wood substrate before and after Ar+ sputtering. No Ag signal was detected, since the amount of silver was below the detection limit of XPS.
